# Transurethral Drainage of Prostatic Abscess: Points of Technique

**DOI:** 10.5812/numonthly.3690

**Published:** 2012-03-01

**Authors:** Mohamed El-Shazly, Nawaf El- Enzy, Khaled El-Enzy, Encho Yordanov, Badawy Hathout, Adel Allam

**Affiliations:** 1 Farwaniya Hospital, Kuwait

**Keywords:** Prostate, Transurethral, Drainage, Methods

## Abstract

**Background:**

The incidence of prostatic abscess (PA) has markedly declined with the widespread use of antibiotics and the decreasing incidence of urethral gonococcal infections.

**Objectives:**

To evaluate different treatment methods for prostatic abscess and to describe technical points that will improve the outcome of transurethral (TUR) drainage of prostatic abscess.

**Patients and Methods:**

We performed a retrospective study of a series of 11 patients diagnosed with prostatic abscess, who were admitted and treated in Farwaniya Hospital, Kuwait, between February 2008 and November 2010. Drainage was indicated when antibiotic therapy did not cause clinical improvement and after prostatic abscess was confirmed by TRUS (Transrectal ultrasonography) and/or CT computed Tomographyscan. TUR drainage was indicated in 7 cases, ultrasound-guided transrectal drainage was performed in 2 cases, and ultrasound-guided perineal drainage was performed in 2 cases.

**Results:**

All patients that underwent TUR-drainage had successful outcomes, without the need of secondary treatment or further surgery.

**Conclusions:**

TUR drainage of a prostatic abscess increases the likelihood of a successful outcome and lowers the incidence of treatment failure or repeated surgery. Less invasive treatment, with perineal or transrectal aspiration, may be preferred as a primary treatment in relatively young patients with localized abscess cavities.

## 1. Background

The incidence of prostatic abscess (PA) has declined markedly with the widespread use of antibiotics and the decreasing incidence of urethral gonococcal infections. Predisposing factors for PA include an indwelling catheter, instrumentation of the lower urinary tract, bladder outlet obstruction, acute and chronic bacterial prostatitis, chronic renal failure, hemodialysis, diabetes mellitus, cirrhosis, and, more recently, acquired immunodeficiency syndrome (AIDS) (1-3). Before the advent of modern antibiotic therapy, 75% of prostatic abscesses were attributable to gonococcus, and the mortality rate was between 6% and 30%. Evidence from the literature indicates that prostatic abscess is diagnosed in only 0.2% of patients with urologic symptoms and in 0.5%–2.5% of patients hospitalized for prostatic symptoms (4-7).

## 2. Objectives

To evaluate different treatment methods for prostatic abscess and to describe technical points that will improve the outcome of TUR drainage of prostatic abscess.

## 3. Patients and Methods

We retrospectively studied a series of 11 patients diagnosed with prostatic abscess, who were admitted and treated in Farwaniya Hospital, Kuwait, between February 2008 and November 2010, using data collected from medical records. The ages of the patients ranged from 43 to 67 years old (mean age, 51) Table 1. All patients had risk factors. Nine patients had diabetes and 2 were receiving hemodialysis. All were initially treated with parenteral antibiotics. Drainage was indicated after there was no clinical improvement with antibiotic therapy and after the diagnosis of prostatic abscess was confirmed. TUR drainage was indicated in 7 cases, ultrasound-guided transrectal drainage was performed in 2 cases, and ultrasound-guided perineal drainage was performed in 2 cases.

**Table 1:** Demographics and Operative Data

**Patients**	**Age,y**	**Pathogens**	**CT^a^Performed**	**Method of Drainage**
1	62	*S. aureus*	Yes	Perineal
2	67	*E. coli*	No	Transrectal
3	43	*S. aureus *	Yes	TUR^a^
4	52	*E. coli*	No	TUR
5	45	*Klebsiella pneumoniae *	Yes	Perineal
6	51	*E. coli*	Yes	TUR
7	48	*E. coli*	Yes	TUR
8	56	*Klebsiella pneumoniae *	No	Transrectal
9	44	*S. aureus*	Yes	TUR
10	53	*E. coli*	Yes	TUR
11	46	*E. coli*	No	TUR

^a^Abbreviations: CT, computed tomography; TUR, transurethral

In this article, we describe some technical points of TUR drainage of prostatic abscess that will help to accurately identify the site of abscess cavity, minimize unneeded resection (TURP), and improve the drainage.

### 3.1. Technique

To prevent septicemia, TUR drainage is performed during preoperative systemic parenteral antibiotic administration and after injection of single doses of cephalosporin and metronidazole. During TUR drainage of prostatic abscess, the site of the abscess is not easy to detect, as it is usually deep-seated in the transitional zone. The site of the abscess cavity can be pre-operatively anticipated with the findings from digital rectal examination, transrectal ultrasonography, and CT scans. Additionally, the release of pus to the prostatic urethra, by intra-operative prostatic massage, can indicate the site of the abscess. Another method is to induce pus release to the prostatic urethra by creating several incisions with a Collings knife in the expected site of the abscess, thus avoiding excessive resection of prostatic tissues. Once the site of the abscess has been localized, proper deroofing of the cavity is performed by resection of prostatic tissues around the cavity’s mouth. In some cases, to ensure complete drainage of the abscess, the thick pus must be milked out by prostatic massage.

## 4. Results

All patients had fever, which was accompanied by urine retention in 8 cases of our series. Three patients presented with severe lower urinary tract symptoms. Six patients had perianal or perineal pain. Ten patients had leukocytosis, while 1 immunocompromised patient on hemodialysis showed a normal leucocyte count.

**Figure 1:**
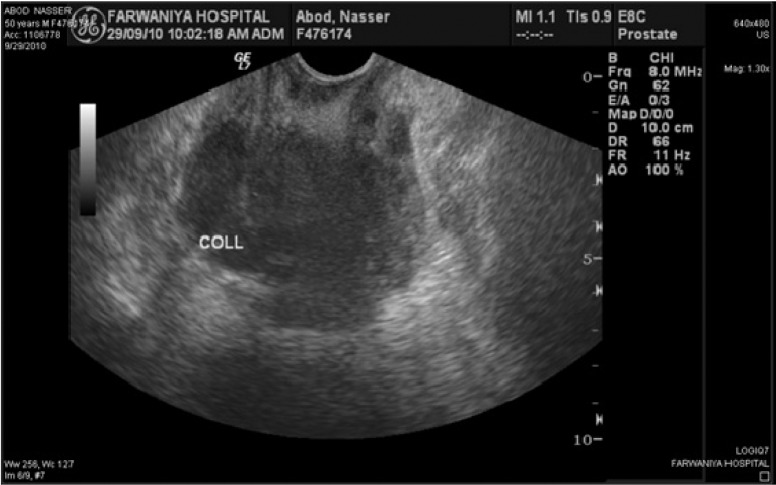
TRUS Showing Abscess Cavity

**Figure 2:**
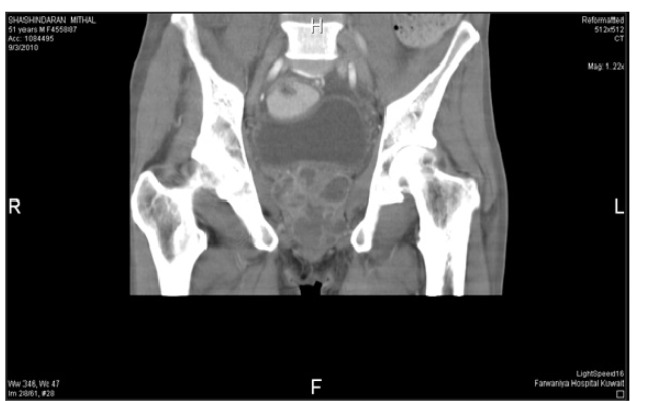
CT Scan Showing Multiple Diffuse Abscesses Within the Prostate

In 10 patients, the prostatic abscess was detected by transrectal ultrasound (Figure 1). One patient, who could not tolerate the transrectal probe, was diagnosed by abdominal ultrasonography. Computed tomography (CT) scans confirmed the abscess in 7 patients (Figure 2). CT scans of our series detected 2 cases with an extraprostatic extension to ischiorectal fossa and in 1 case, detected emphysematous prostatitis, a rare condition (8).

All patients were initially treated with empiric parenteral antibiotics during their hospital stays, with 4.5 gram piperacillin plus tazobactam administered intravenously (IV) tds or in some cases, 400 mg ciprofloxacin IV bid and 500 mg metronidazole IV tds. The 2 patients on hemodialysis were prescribed 1 gram ceftriaxone od, as it is safe with renal failure. Seven patients received TUR drainage of their abscesses (4 received it as primary treatment and 3 as secondary treatment after failed aspiration). The abscesses of 2 patients were perineally drained, due to the periprostatic extension of the abscess to the ischiorectal fossa and perineum. Two patients received transrectal drainage due to posterior abscess bulging into the space between the prostate and the rectum. The most frequently identified pathogen was Escherichia coli, which was found in 6 cases, followed by Staphylococcus aureus in 3 cases and Klebsiella pneumoniae in 2 cases. There were no tuberculosis cases in this series. All patients were negative for human immunodeficiency virus (HIV).

In 5 out of the 7 patients who underwent TUR drainage, the abscess cavity was located on the apical part of the prostate, close to the verumontenum. Resection was confined to the apical part of the abscess’s location, and resection of the whole lobe and bladder neck was avoided to prevent the risk of retrograde ejaculation. In late follow-up of the 7 patients that received TUR drainage, only 2 of the patients had retrograde ejaculation. Successful drainage was achieved in all TUR drainage cases, without the need of secondary treatment or further surgery.

## 5. Discussion

Prostatic abscess is an infrequent clinical occurrence that can be difficult to diagnose, due to its presentation with non-specific symptoms. Symptoms and clinical findings of prostatic abscess are extremely variable. Fever and painful and frequent micturition are common with acute prostatitis. A prostatic abscess may progress to spontaneous fistulization into the urinary bladder, prostatic urethra, rectum, or perineum. In some cases, it can lead to sepsis and death (9-11). Thus, both an accurate diagnosis and an efficient treatment are required. Most published data regarding prostatic abscesses are case reports, and there is no standardization of the diagnostic and therapeutic routines. In review articles, the summary of several individual experiences permits delineating some lines of action for prostatic abscess (1, 2).

The diagnostic method of choice, which assists in the treatment and follow-up of patients with prostatic abscess, is transrectal ultrasonography of the prostate. The most common finding is the presence of 1 or more hypoechoic areas, which contain thick pus primarily in the transition zone and in the central zone of the prostate, and which are permeated by hyperechogenic areas and distortion of the anatomy of the gland (9). This finding, observed in all cases in this study for which the examination was performed, supports the use of transrectal ultrasonography for the diagnosis of prostatic abscess, for detection of extraprostatic collections, and to detect gas in the fluid (emphysematous prostatitis) (9). Transrectal sonography usually underestimates the real periglandular extension of the abscess (9-12). Detecting periprostatic extension, particularly to the ischiorectal fossa and perineum, is important, as perineal drainage is easier and expected to be more successful than the TUR drainage, which we observed in case number 5.

Treatment of prostatic abscess is implied in parenteral broad-spectrum antibiotic administration and abscess drainage. This may be performed by transrectal or transperineal ultrasound-guided, digital-guided puncture/ drainage by the perineal route, transurethral incision of the prostate, TURP, or open perineal drainage. All methods have been reported to be safe and effective. Recent findings suggest that less invasive treatment by ultrasound-guided percutaneous or transrectal drainage is preferred to TUR drainage because it can be performed under local anesthesia or sedation and repeated if necessary. Less invasive methods also have a lower risk of complications, in particular, possible retrograde ejaculation after TUR drainage in relatively young patients (13-15).

TUR drainage should be reserved for cases with multiple and diffuse prostatic abscesses or when aspiration does not show complete resolution of the fluid collection (9). In this series, 7 patients (63.6%) received TUR drainage of their abscesses (4 had diffuse multiple abscesses and were not amenable for aspiration, and 3 cases, unsuccessfully treated with aspiration, were given TUR drainage as the secondary treatment). Using some procedural points, which have been described in the Materials and Methods section, to accurately localize the abscess cavity during TUR drainage is very important, particularly in young patients, in order to limit TUR drainage to the abscess cavity, and thus avoid the occurrence of retrograde ejaculation after complete TURP.

Prostatic abscess should be suspected in patients presenting with fever and persistent lower urinary tract symptoms that do not respond to antibiotics. Less invasive treatments, such as perineal or transrectal aspiration, are preferred as the primary treatment in relatively young patients with localized abscess cavities. TUR drainage is recommended in cases with diffuse, large abscess cavities or after failed aspiration. Several techniques, which we have described above, are very helpful during TUR drainage in minimizing resection and avoiding retrograde ejaculation in relatively young patients. TUR drainage of prostatic abscesses has a high likelihood of success and a low incidence of treatment failure or further surgery.
